# Stable carbon isotope as a signal index for monitoring grassland degradation

**DOI:** 10.1038/srep31399

**Published:** 2016-08-16

**Authors:** Hongyun Yao, Andreas Wilkes, Guodong Zhu, Hongdan Zhang, Xiaojuan Liu, Dan Ding, Xiajie Zhai, Shiming Tang, Qing Chen, Yujuan Zhang, Ding Huang, Chengjie Wang

**Affiliations:** 1 College of Ecology and Environmental Science, Inner Mongolia Agricultural University, Hohhot 010019, Inner Mongolia, China; 2 Values for Development Limited, Bury St Edmunds, IP33 3EQ, UK; 3 Department of Grassland Science, China Agricultural University, Beijing 100193, China; 4 Tianjin Key Laboratory of Water Resources and Environment, Tianjin Normal University, Tianjin 300387, China; 5 Institute of Grassland Science, Chinese Academy of Agricultural Science, Hohhot 010010, China

## Abstract

Grassland degradation due to overgrazing is common in many areas of the world. This study analyzed the potential of the stable carbon isotope (*δ*^13^C) value as a structural microcosmic index to monitor processes of grassland degradation. The *δ*^13^C values of plant leaves, roots and soils in non-grazed (NG) and over-grazed (OG) grassland were measured from samples collected from the seven types of grassland in China. We found that the leaf *δ*^13^C values of palatable species (*δ*^13^C_leaf_) and root *δ*^13^C values (*δ*^13^C_root_) in OG grasslands were reduced compared with those from NG grasslands. Furthermore, the *δ*^13^C_leaf_ and *δ*^13^C_soil_ were positive correlation with elevation and latitude, *δ*^13^C_root_ was negative correlation with them at high altitude (3000~5000m), and *δ*^13^C_root_ and *δ*^13^C_soil_ were negative correlation with them at low altitude (0~2000m), respectively. Consequently, tracing of the *δ*^13^C variations in grassland ecosystem can provide a powerful tool to evaluate the degree of grassland degradation.

Due to a combination of human impacts and climatic changes, grasslands have suffered substantial degradation during past decades in many areas of the world, including in arid and semi-arid regions of China^1^,^2^,^3^. To keep track of such changes and predict further degradation, conventional indicators such as plant height, cover, density and biomass and physical and chemical characteristics of soil are used to monitor rangeland degradation^4^,^5^. Here, *δ*^13^C values are evaluated as a microcosmic index for monitoring processes of grassland degradation.

Grazing disturbance not only reduced the *δ*^13^C_leaf_ value of palatable species such as annuals, perennial grasses and legume (Table S1, Fig. 1a,b), but also converted the photosynthetic pathways of three native species from C_3_ to C_4_ (Table S2). The underlying mechanism is most likely that the degraded sites experienced decrease of both leaf area index and the ratio of leaf blades to stem^6^, leading to a decrease of the net photosynthetic rate^3^ and an increase of the intercellular carbon dioxide concentration (*Ci*) (Table S3). As adjacent NG and OG plots in one site were assumed to be equal in ambient carbon dioxide concentration (*C*_*a*_), observed changes in *δ*^13^C_leaf_ can be described by the follow equation^7^,


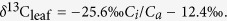


In addition, the *δ*^13^C_leaf_ values were positive correlation with elevation and latitude, while negative correlation with longitude respectively (Fig. S1(a~c)), which may be due to atmospheric variations in *δ*^13^C constituting only a minor portion of the observed differences in plant tissue composition, and the elevational differences in carbon isotope composition appearing to reflect real differences in discrimination by plants^8^. Moreover plants *δ*^13^C content at high altitude depends largely on hours with high radiation^9^,^10^.

Values of *δ*^13^C_root_ were significantly increased in most degraded grasslands and decreased in others (Fig. S2). In addition, *δ*^13^C_root_ had a similar value to *δ*^13^C_leaf_ in NG sites, but showed an increase and then a decrease along degradation gradient (Fig. S3). The *δ*^13^C_root_ was significantly increased in most grasslands types, this was possibly because increasing root/shoot ratio and biomass allocation to the roots was an important adaptive response of plants to grazing, reflecting that a high proportion of root biomass in total biomass can enhance the capacity of plant to tolerate environmental stresses and buffer external disturbances^5^,^11^. Plant transport dynamics, such as temporal changes in C allocation and metabolic processes along the transport pathways in the phloem of stems, determined the coupling of the isotope signals above and below ground^9^. Since the metabolites were enriched in ^13^C with reference to photosynthetic products, the large amount of stem reduction and diminution of individual plant size in OG plots shortened the transport and metabolite pathway, leading to the relative ^13^C depletion of metabolites transported to roots^9^. Mechanisms for *δ*^13^C_root_ enrichment in lightly grazed plots (LG) and depletion in OG plots may be that root in LG plot (Fig. S3) were still able to buffer animal disturbances (intermediate disturbance hypothesis). However, heavy grazing significantly removed leaf and stem biomass, resulting in a reduction in *δ*^13^C_root_^4^. The differences in isotopic signatures between plant organs are consistent with the notion that the ^13^C enrichment of particular compounds occurs in a basipetal direction^12^. The *δ*^13^C_root_ values showed different correlation with geographic factors at high and all altitude (Fig. S1(d)~(f,m)~(o)), especially decreased significantly with an increase of elevation and increased with an increase of latitude and longitude at low elevation (Fig. S1(j,k)), which generated a well performed multiple regression model (Fig. 2a,b).

Data from seven types of grassland show that after grazing, *δ*^13^C_soil_ rose in some and fell in other grassland types (Fig. 3). These contrasting trends in *δ*^13^C_soil_ after grazing may be related to the significant difference in the interaction between non-grazed, over-grazed treatments and region (Fig. 3, p < 0.0001), indicating that different grassland types have different tolerance to the same grazing intensity. The model for factors affecting *δ*^13^C_soil_ was developed as follows (Fig. 3):


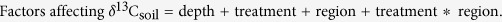


Heavy grazing may disrupt the structure of soil aggregates and surface crust because of livestock trampling, which increases the occurrence of medium and small particle-sized organic matter, induces soil organic matter to decompose more rapidly and leads to the soil being susceptible to water and wind erosion^13^. We considered that this situation would lead to a better moisture conditions in NG plots compared to OG ones, A much larger amount of soil moisture was evaporated in the grazing plots, probably because of less litter coverage on the soil surface^3^. It has been proved that better moisture conditions favor more open stomata and the preferential uptake of ^12^C over ^13^C^14^. Based on the G. D. Farquhar theory^7^, the developing application^15^, and the relative equal climate conditions at our adjacent NG and OG plots, we reasoned that *δ*^13^C_leaf_ was determined indirectly by the ratio of net photosynthetic rate to stomata1 conductance (*A*/*g*). The *δ*^13^C was enriched along the plant axis downward, leading to an increase of *δ*^13^C_soil_^9^,^16^. Grazing induced the relative drought stress, favor more close stomata, leading to *g* decreasing and *A*/*g* variation. Consequently, *δ*^13^C_soil_ was relative depleted or enriched at OG plots (Fig. 3). Moreover, drought decreases the contribution of recently assimilated C to soil CO_2_ efflux and can increase the residence time of recently assimilated C in leaf biomass^17^. Up to 40% of photosynthates are exudated by roots and are rapidly respired or invested in biomass by rhizosphere microorganisms^18^. Since root carbon storage might supply autotrophic respiration and enable respiration rates temporarily, the respiration of microbes around the rhizosphere might decline more rapidly after root is removed by grazers, thus decreasing belowground biomass^14^,^19^. Values of *δ*^13^C_soil_ in the seven OG plots showed a higher correlation with elevation and latitude (*R*^*2*^ = 0.810, *p* < 0.0001) than *δ*^13^C_soil_ in the seven NG plots (*R*^*2*^ = 0.576, *p* < 0.0001). This possibly indicates that *δ*^13^C_soil_ was more related to geographic (Fig. S1(g)~(i),(o)), and even indirect climatic factors^20^, when grassland is degraded (Fig. 2c,d).

Although the trends in *δ*^13^C_root_ and *δ*^13^C_soil_ were not in the same direction after grazing (Fig. 3 and S2), the difference interval of *δ*^13^C_root_ and *δ*^13^C_soil_ in OG plots Δ_r-sOG_ (Δ_r-sOG_ = Δ_root-soilOG_ = *δ*^13^C_rootOG_ − *δ*^13^C_soilOG_) was little changed when compared to Δ_r-sNG_ (Δ_r-sNG_ = Δ_root-soil NG_ = *δ*^13^C_rootNG_ − *δ*^13^C_soilNG_) (Fig. 4). This indicates that despite the complex processes of belowground carbon allocation, the C isotopic signature of soil and roots after grazing (Δ_r-sOG_ and Δ_r-sNG_) is a promising approach to partitioning C sources of soil respiration, monitoring belowground biological activity, and potentially identifying and quantifying the mechanisms of C stabilization and release^9^. Previous studies proved that carbon isotope indicators can provide information on diffuse air pollution^21^, reconstruct the past climate change using the *δ*^13^C of buried soils^22^, and reconstruct palaeoclimatic of lake throughflow using isotope data of plant macroremains and authigenic carbonates^23^, and reconstruct *Phyllocladus* using *δ*^13^C in a range of New Zealand proxies and macrofossils^24^, and so on. This study provided another possible idea of degraded grassland reconstructions, isotope data (Fig. 4) can provide complementary information to reconstruct and analyse the environmental perturbations of grazers. The isotope approach more or less contributes to construct more detailed global isotopic gradients in grassland when combined with *δ*^13^C, *δ*^18^O and *δ*^15^N of other species and other field (forest and ocean) worldwide^24^.

Overall, the microcosmic index *δ*^13^C of leafs and roots allows an integrated understanding of the process of grassland degradation, and can be a powerful tool permitting both tracing of C molecules and an integrated view of biological processes in the degradation of ecosystems across space and time.

## Methods

### Survey of representative grassland regions

The experiment selected seven types of grasslands in the Eurasian grassland zone: plain meadow, meadow grassland, typical grassland, desert grassland, temperate marsh grassland, alpine meadow and mountain desert grassland. The entire zone extends from northeast to southwest, stretching latitudinally over 16° 3′ and longitudinally over 36°11′, with an elevation range of 4200 m and a distance of nearly 3000 km across the zone. These grasslands have been over-grazed for nearly 40 years. More detailed information on the sites is provided in the Supplementary Information.

### Experimental design and sampling of the surveyed grassland areas

Different land use types in each site were selected as study plots. These were fenced in the 1970s as non-grazed plots (NG) and severely degraded grassland that had been over-grazed (i.e. OG plots). Sites were sampled in August 2014 by randomly selecting three blocks in each plot (Fig. S4).

### Method for *δ* ^13^C analysis

The *δ*^13^C analysis used a cavity ring down spectrometer (CRDS) (Picarro G2201-I *Picarro, Inc*. Santa Clara, CA, USA) provided by the Isotope Analysis Laboratory of Inner Mongolia Agriculture University. The leaves intercellular CO_2_ concentration (*Ci*) of leaves was measured using an *LI-6400* Portable Photosynthetic System (*Li-Cor*, Lincoln, NE, USA).

### Data calculation

Data were analyzed with SPSS version 20.0 (SPSS for Windows, Chicago, IL, USA), and figures were charted with SigmaPlot version 12.5 software. Correlation analysis was performed to investigate relationships between *δ*^13^C values and geographical factors. Univariate analysis using a general linear model was performed on *δ*^13^C_soil_ with depth, region and grazing treatments, and on *δ*^13^C_root_ with region and grazing treatments. The same procedure was also used on the difference interval of *δ*^13^C_root_ and *δ*^13^C_soil_. Univariate and multivariate linear regression analysis and nonlinear regression were performed on *δ*^13^C values and geographical factors. Means were tested with Tukey’s test when treatment effects were considered statistically significant (P < 0.05).

## Additional Information

**How to cite this article**: Yao, H. *et al*. Stable carbon isotope as a signal index for monitoring grassland degradation. *Sci. Rep.* **6**, 31399; doi: 10.1038/srep31399 (2016).

## Supplementary Material

Supplementary Information

## Figures and Tables

**Figure 1:**
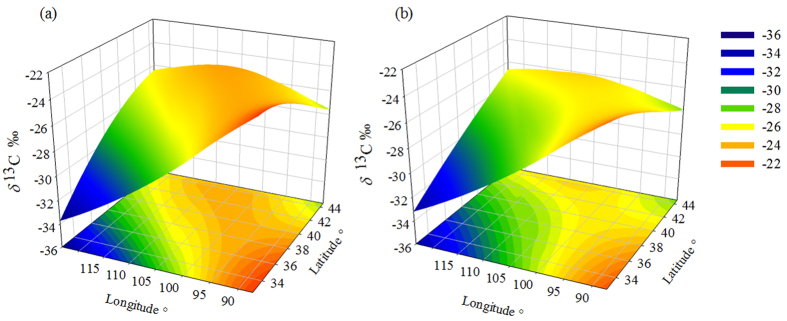
The *δ*^13^C_leaf_ values in non-grazed and over-grazed sites in the seven types of grassland. Images show latitudinal and longitudinal trends in *δ*^13^C_leaf_ variation. Panel (**a**) represents *δ*^13^C_leaf_ on non-grazed (NG) grasslands, while panel (**b**) shows *δ*^13^C_leaf_ of the same species on over-grazed grasslands (OG).

**Figure 2:**
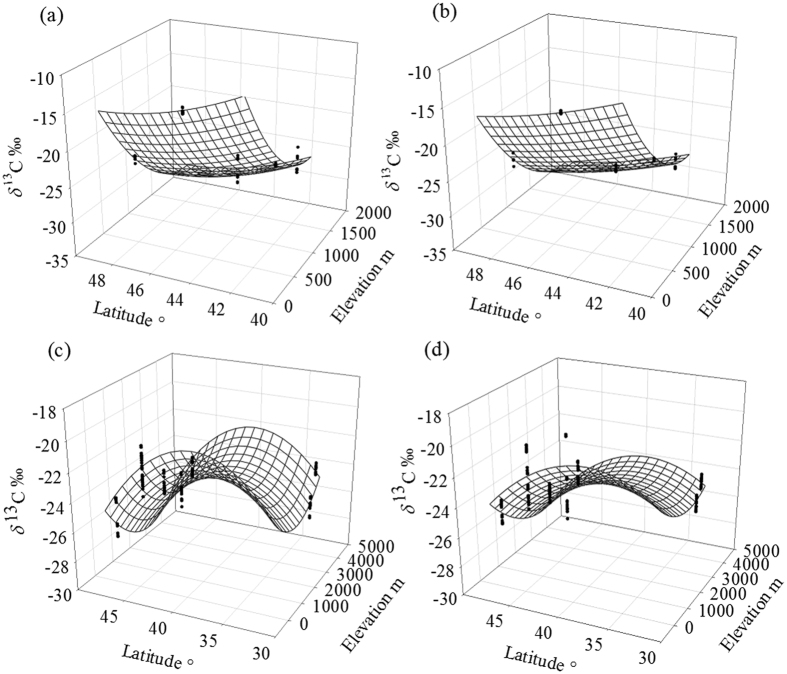
The multiple regression of *δ*^13^C_root_ (at low elevation) and *δ*^13^C_soil_ (at all elevations) with latitude and elevation. Panel (**a**) shows the regression of *δ*^13^C_root_ (at low elevation <2000m) in NG grasslands (*R*^2^ = 0.972 *p* < 0.0001 n = 45), while panel (**b**) shows the *δ*^13^C_root_ regression in OG grasslands (*R*^2^* = *0.993 *p < *0.0001 n = 45). Panel (**c**) shows the *δ*^13^C_soil_ regression at all elevations (0~5000m) regression in NG grasslands (*R*^2^* = *0.810 *p < *0.0001 n = 188), while panel (**d**) shows the *δ*^13^C_soil_ regression in OG grasslands at all elevations (*R*^2^* = *0.576 *p < *0.0001 n = 188).

**Figure 3:**
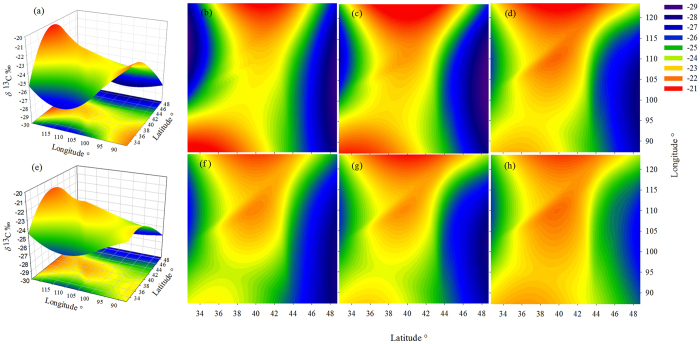
The *δ*^13^C_soil_ values at three depth intervals in non-grazed and over-grazed sites in seven grassland types. Panel (**a**) shows the latitudinal and longitudinal trend in *δ*^13^C_soil_ variation in all layers on non-grazed (NG) grasslands, and panel (**e**) shows the latitudinal and longitudinal trend in *δ*^13^C_soil_ variation in all layers on over-grazed (OG) grasslands. Panels (**b–d**) show *δ*^13^C_soil_ variation at sampling depths of 0–5 cm, 5–10 cm and 10–15 cm, respectively, on NG grasslands, while panels (**f–h**) show variation at the same depth in OG sites. The p-value of depth, treatment and region are 0.001, 0.035 and 0.000 respectively. The p-value of interaction between treatment and region is 0.0001. In the model developed, the interaction of treatment, depth and region is taken into consideration. Grazing lead to *δ*^13^C_soil_ increase at middle latitude and longitude sites, and decrease at high and low latitudes and longitudes at 0–5 cm and 5–10 cm depth, while the opposite trend occurs at on 10–15 cm depth. Grazing disturbed *δ*^13^C_soil_ of the surface soil more than it disturbed of deeper soil layers.

**Figure 4:**
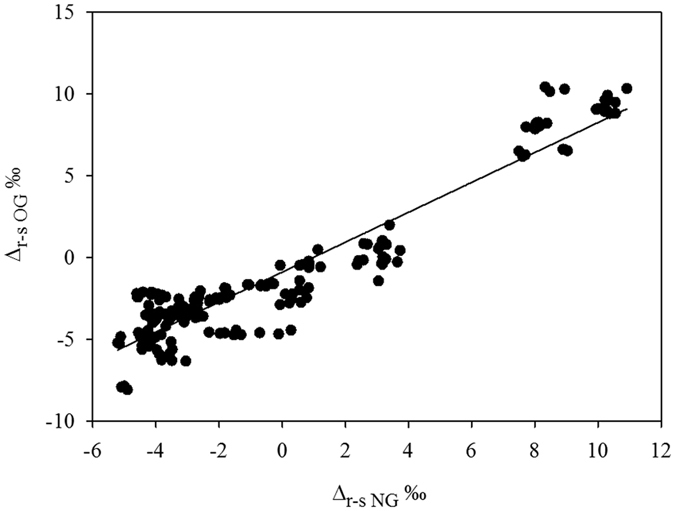
The linear regression of the difference interval between *δ*^13^C_root_ and *δ*^13^C_soil_ at over-grazed and non-grazed sites. Δ_r-sNG_ represents the difference interval of *δ*^13^C_root_ and *δ*^13^C_soil_ in non-grazed plots (Where Δ_r-sNG_ = Δ_root-soil NG_ = *δ*^13^C_rootNG_ − *δ*^13^C_soilNG_ (‰)), Δ_r-sOG_ represent the difference of *δ*^13^C_root_ and *δ*^13^C_soil_ in over-grazed plots (Δ_r-sNG_ = Δ_root-soil NG_ = *δ*^13^C_rootNG_ − *δ*^13^C_soilNG_ (‰)).
