# Supplementary material for: Stable carbon isotope as a signal index for monitoring grassland degradation
Source: Sci Rep. 2016 Aug 16;6:31399. doi: 10.1038/srep31399 (PMC4985657; doi:10.1038/srep31399)
Supplement: Supplementary Information [file srep31399-s1.pdf]

Stable carbon isotope as a signal index for monitoring grassland degradation

Hongyun Yao[1], Andreas Wilkes[2], Guodong Zhu[1], Hongdan Zhang[1], Xiaojuan Liu[1], Dan Ding[1], Xiajie Zhai[3], Shiming Tang[3], Qing Chen[4], Yujuan Zhang[5], Ding Huang[2], Chengjie Wang[1]

[1]College of Ecology and Environmental Science，Inner Mongolia Agricultural University，Hohhot 010019，Inner Mongolia, China. [2]Values for Development Limited, Bury St Edmunds, IP33 3EQ, UK. [3]Department of Grassland Science, China Agricultural University, Beijing 100193, China. [4]Tianjin Key Laboratory of Water Resources and Environment, Tianjin Normal University, Tianjin 300387, China. [5]Institute of Grassland Science, Chinese Academy of Agricultural Science, Hohhot 010010, China.

Correspondence and requests for materials should be addressed to Chengjie Wang (nmgcjwang3@163.com) or to Ding Huang (huangding@263.net).

Supplementary information

Site description

Changling County in Jilin, Ewenke Banner in Inner Mongolia, Xilinhot in Inner Mongolia, Siziwang Banner in Inner Mongolia, Hongyuan County in Sichuan, Yushu County in Qinghai and Fuhai County in Xinjiang were selected as representative sites where the Chinese government's Grassland Retirement Program has been implemented. Each representative site had one overgrazed areas (OG) with one adjacent non-grazed areas (NG) as a control, which were not less than 600 ha (2 km×3 km) in any one site.

Plain meadow: The plain meadow is located in Yaojingzi ranch in Changling county, and is the site of the Songnen Grassland Ecosystem Research Station of Northeast Normal University, Jilin (44°35' N, 123°30' E). The site has an elevation of 140 m and is in a northern temperate continental monsoon climate zone, with a frost-free period of 140~150 days. Annual average temperature is 5.2℃. Average annual precipitation is approximately 430 mm, of which nearly 75% falls between July and August. The amount of solar radiation is approximately 540 kwh/m^2. Dominant soil types are saline and alkalize meadow soil,with a pH of 7.5~9. The grassland is dominated by *Leymus chinensis* and *Phragmites australis*,and common grazing livestock are northeast fine wool sheep and Simmental cattle.

Meadow steppe: The meadow steppe is located in Ewenke Banner, Inner Mongolia (48°43' N, 119°55' E). The site has an elevation of 740 m and and is in a temperate semi-arid and continental climate zone. Annual average temperature is -6.56~4.95℃ with a frost-free period of 95-110 day . Annual mean precipitation is 247.6mm, mostly falling as rain between May and August. The amount of solar radiation is approximately 500 kwh/m^2. The soil type is dark Kastanozem and Chernozem. The constructive species are *Stipa baicalensis* and *Carex pediformis*, and the main grazing livestock are Wuzhumuqin sheep and Mongolian cattle.

Typical steppe: The typical steppe is located in Maodeng ranch, Xilinhot, Inner Mongolia (44°09' N, 116°20' E)., where Inner Mongolia University Grassland Ecology Research Base is located. The site has an elevation of 1100 m and the climate is a semi-arid continental temperate climate with dry springs and moist summers. The mean annual temperature is 2℃, with a herbage

growing period of 150d. The amount of radiation is approximately 540 kwh/m^2 and average annual precipitation is 350 mm, mostly concentrated between June and August. Chestnut and dark chestnut soils are the zonal soil types. The grassland is dominated by *Stipa grandis* and *Leymus chinensis*, which are mainly grazed by Mongolian sheep and goats.

Desert steppe: The desert steppe is located in Siziwang Banner in the mid-west of Inner Mongolia (41°47' N, 111°53' E). The site has an elevation of 1450 m and is in a temperate continental climate, characterized by a short growing season and long cold winter. The mean annual temperature is 3.4℃ with frost-free period of 175 days. The average annual precipitation is approximately 280 mm, of which nearly 75% falls during June through September. The amount of radiation is approximately 620 kwh/m^2. The dominant soil types are Kastanozem and Brown Chernozem with a loamy sand texture. The grassland is dominated by *Stipa breviflora Griseb* and *Artemisia frigida Willd*. The main grazing livestock are Mongolian sheep and the Mongolian cattle.

Alpine meadow: The alpine meadow is located in a state-owned farm in Yushu county, as the site of Sanjiangyuan Wildlife Observatory of Qinghai University, Qinghai(33°6' N, 96°51'E). The site has an elevation of 4300 m and an alpine climate. The annual average temperature is -5.6~3.8℃ with a herbage growing period of 156 days and no absolute frost-free period. Annual mean precipitation is 560mm. The amount of radiation is approximately 420 kwh/m^2. The soil type is dark Kastanozem and alpine meadow soil. The constructive species are *Kobresia pygmea* and *Kobresia humilis*. The main livestock are Tibetan sheep and yaks.

Temperate marsh steppe: The temperate marsh steppe is located in Hongyuan county, Aba Tibetan Autonomous Prefecture in Sichuan, at the eastern edge of the Qinghai-Tibet Plateau （32°40′ N, 102°18′E). The elevation of the site is 3487~3581m and the climate is a cold temperate continental plateau monsoon climate. Annual average temperature is 1.1℃ with no absolute frost-free period. Annual mean precipitation is 752.4 mm. The amount of radiation is approximately 660 kwh/m^2. The main soil types are subalpine meadow soil and meadow marsh soil. The constructive species are *Cyperaceae* and herbs. The vegetation type are subalpine meadow and marshy meadow.

Mountain desert steppe: The mountain desert grassland is located in Yongfeng town, south of Urumuqi, Xinjiang (43°40' N, 87°19'E). The elevation of the site is 1850 m and the climate is a mid-temperate continental arid climate. The annual average temperature in July and August is 25.7℃, while January average temperature is -15.2℃. The absolute frost-free period is 150 days. Annual mean precipitation is 194mm. Spring and autumn seasons are relatively short, while winter and summer are longer with a large diurnal temperature difference. The amount of radiation is approximately 580 kwh/m^2. The soil types are brown soil and gray desert soil. The vegetation types are mountain meadow and mountain desert meadow.

Materials and Methods

Survey Procedure

Location: The latitude and longitude of each study site was recorded at the center point using a GPS and marked with a stake. Transects were set bearing either 120°, 240°, or 360°N (Fig. S4).

Leaves: The leaves of species on 3 transects in each study site were measured. Along each transect 3 plots (50 cm by 50 cm) were clipped at 5, 15, and 25 meters. The leaves collected were growing towards the sun with a strong active photosynthesis, and new growing or ageing leaves were avoid, because they are not suitable to represent average leaf physiological activity. The leaves were sealed in watertight plastic bags immediately after being removed.

The plant tissues were washed five times with deionized water, sundries and then oven-dried at 60℃ for 24 hours in a forced-air oven. Representative parts were ground to 0.25 mm with a ball grinder. The powder was them put into sealed bottles, marking the sample numbers on the bottle bodies and lids corresponding to their names, after which they were stored in a shady and cool place until dry weight was determined.

Roots: At 15 meters from center point along each transect, a quadrat 50cm long and 50cm wide was taken at three layers (0-5, 5-10, and 10-15cm) using a 6 cm diameter soil auger. Each core was divided into depth intervals of 0-5, 5-10 and 10-15cm, and the samples from each location were pooled to give three replicates. Samples were collected with shovels and put into watertight plastic bags after mixing the layers.

The roots were oven-dried at 60℃ for 24 hours after being cleaned with deionized water and cleaned of other materials. Then samples of dried roots approximately 50-200 mg were ground

with mortar and pestle and filtered by passing through a 250 μm stainless steel sieves until the complete sample was ground and homogenized. The powder was then put into sealed bottles, marking the sample numbers on the bottle bodies and lids corresponding to their names, and stored in shady and cool place.

Soil: Samples (100 cm^3) were taken at three layers (0-5, 5-10, and 10-15cm) using a 6 cm diameter soil auger at a point 15 meters from the center point along each transect at the location where leaves and roots were measured. Each layer was sampled evenly across the 70-cm width to obtain 1000-2000 g of soil that was air-dried. Samples at each soil depth from the each quadrat were thoroughly mixed.

The soil pretreatment methods were the same as for roots. After oven-drying and grinding, the soil samples were pretreated with 0.5mol/L HCl in a beaker with a glass rod and stirred every one hour until the bubbles had disappeared. This process was conducted for about 6 hours to remove soil inorganic carbon materials and carbonate. The solution was precipitated for an hour and the supernatant poured out, after which was washed with deionized water 3 to 4 times to remove the unreacted HCl. Finally both the acid-treated and acid-untreated soil samples were put into bottles, which were marked and stored in a shady and cool place.

Measurement: An electronic scale with a precision of one over one million was used. Different samples based on their unique carbon content have their own weight ranges. We weighed plant tissue in the range of 0.650~0.750mg (including leaves and roots), and soils were weighted in the range of 0.780~0.880mg (0-5cm), 13.000~16.000 (5-10cm) and 20.000~27.000 (10-15cm). We used tweezers to place the individual tin capsule (5×9 mm) on the leveled scale, then reset the scale and transferred the sample to the tin capsule with a medicine spoon which was cleaned every time. Capsules were then crimped closed to small round, and put them to the sample plate with individual cell in order. Measurements were undertaken using a Picarro device composed of a Combustion Module (CM), liaison and G2201-*i*.

The intercellular carbon dioxide concentration (*Ci*) was measured with an *LI-6400* Portable Photosynthetic System (*Li-Cor*, Lincoln, NE, USA). *Leymus chinensis (Trin.) Tzvel.* and *Stipa capillata L.* were randomly selected from NG and OG plots in the desert steppe. We measured leaves photosynthetic characteristics from 9:00am to 12:00am in August 2014.

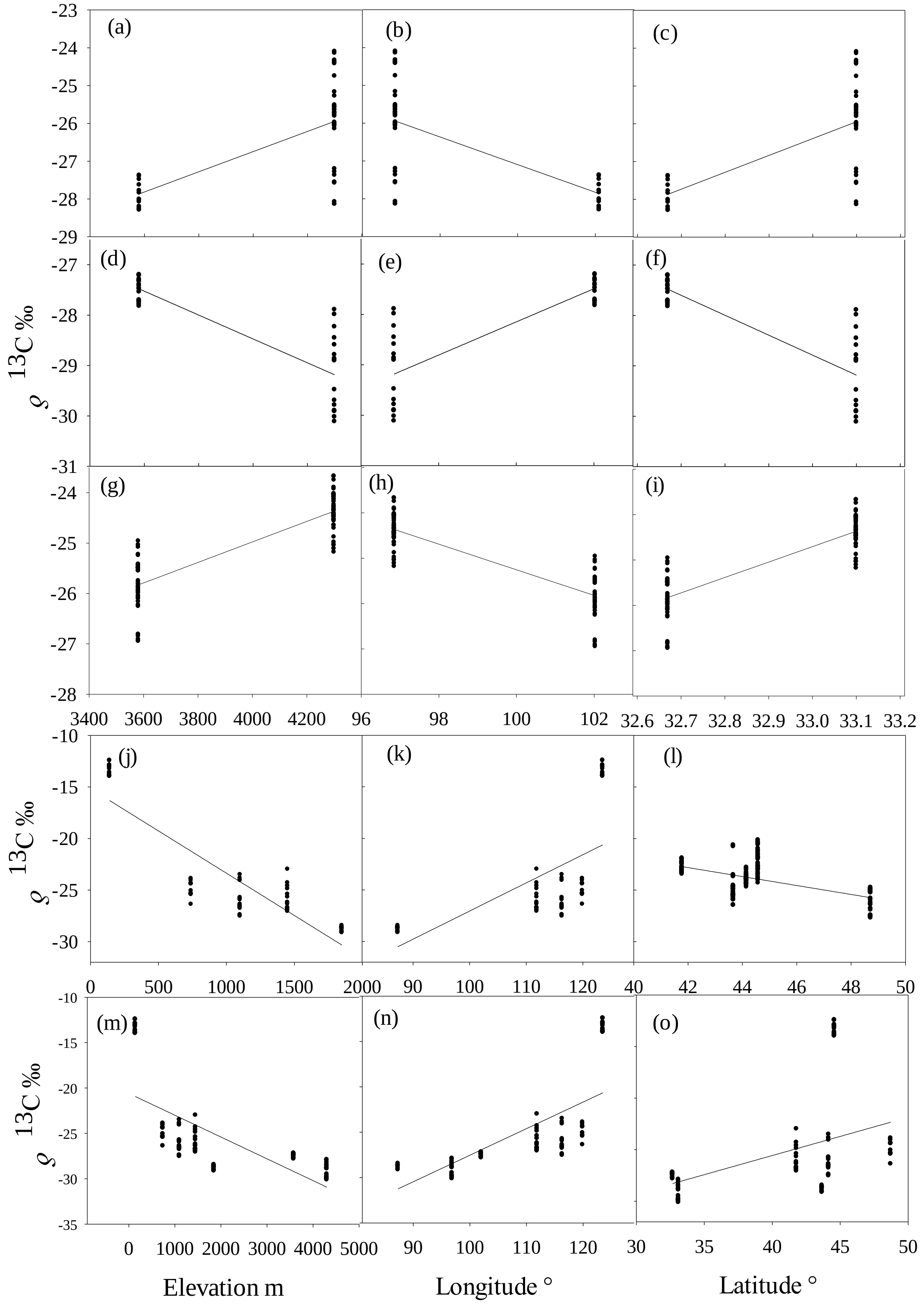

Figure S1 | The correlations between δ^{13}C values and geographic factors.

At high altitude (3000~5000m):

The $\delta^{13}C_{leaf}$ values were positive correlation with elevation (panel (a)) and latitude (panel (c)), and negative correlation with longitude (panel (b)), respectively, the coefficients of correlation (R^2) from (a) to (c) are 0.405. The $\delta^{13}C_{root}$ values were negative correlation with elevation (panel (d)) and latitude (panel (f)), and positive correlation with longitude (panel (e)), respectively, the coefficients of correlation (R^2) from (d) to (f) are 0.714. The $\delta^{13}C_{soil}$ values were positive correlation with elevation (panel (g)) and latitude (panel (i)), and negative correlation with longitude (panel (h)), respectively, the coefficients of correlation (R^2) from (g) to (i) are 0.752.

At low altitude (0~2000m):

The $\delta^{13}C_{root}$ values were negative correlation with elevation (panel (j), R^2=0.779) and positive correlation with longitude (panel (k), R^2=0.412), respectively. The $\delta^{13}C_{soil}$ values were negative correlation with longitude (panel (l), R^2=0.340).

At all altitude (0~5000m):

The $\delta^{13}C_{root}$ values were negative correlation with elevation (panel (m), R^2=0.445) and positive correlation with longitude (panel (n), R^2=0.502) and latitude (panel (o), R^2=0.169), respectively.

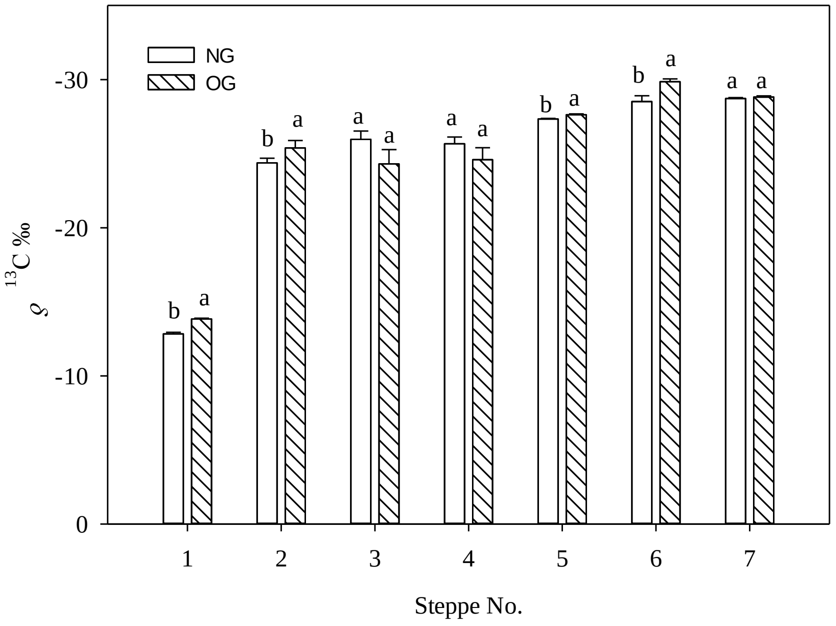

Figure S2 | The $\delta^{13}C_{root}$ values from paired non-grazed and overgrazed treatments in seven grassland types. Different letters indicate significant difference among the average of three plots (Tukey test, $p<0.05$). The grassland types are (1) plain meadow, (2) meadow steppe, (3) typical steppe, (4) desert steppe, (5) temperate marsh steppe, (6) alpine meadow, (7) mountain desert steppe, respectively. The p-value of region, treatment and the interaction between treatment and region are 0.0001.

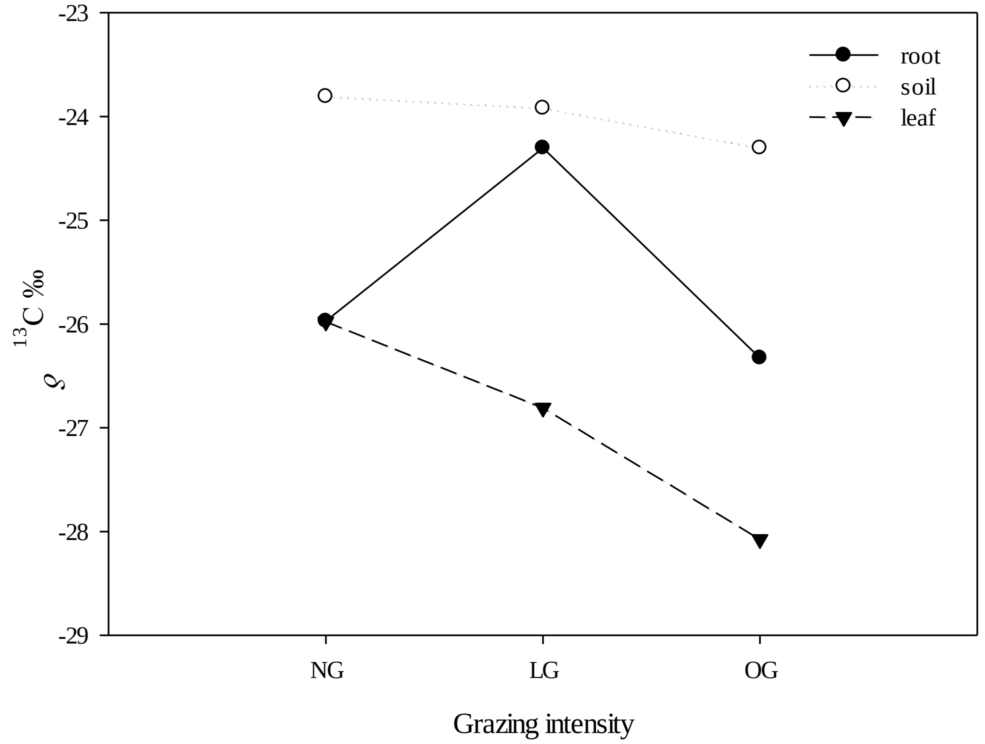

Figure S3 | The variation trend of leaf, root and soil δ^{13}C values along a grazing intensity. NG, LG and OG represent non-grazed, lightly grazed and over-grazed plots, corresponding with a degradation gradient.

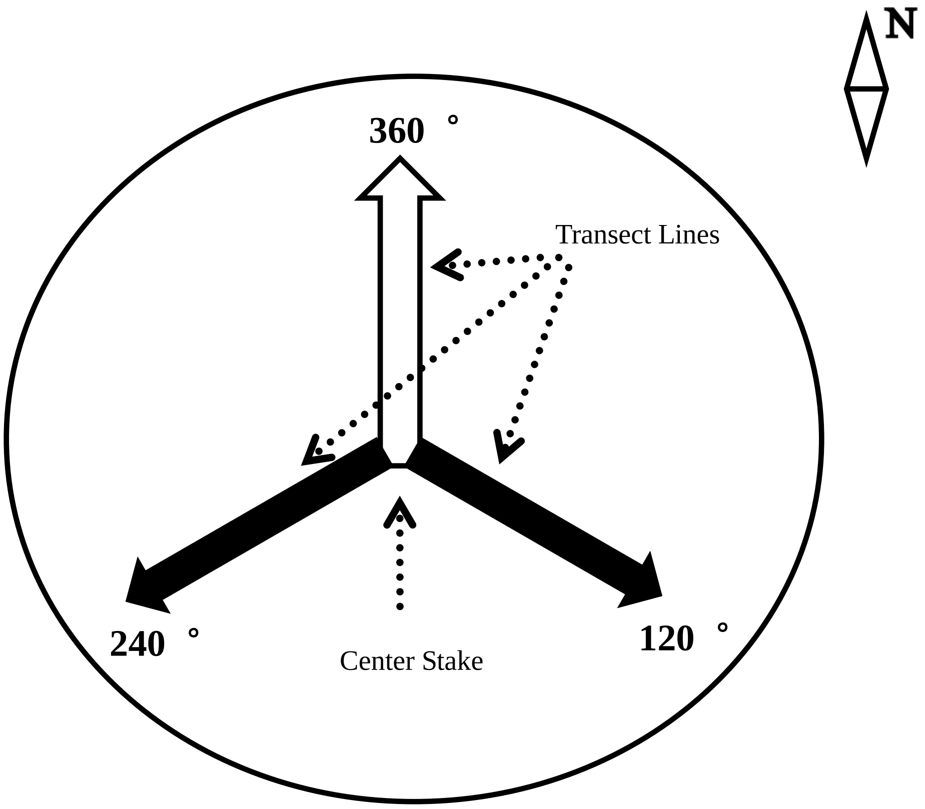

Figure S4 | Diagram of each sampling site.

Table S1 | The carbon isotope composition (δ^{13}C value) and descriptive characteristics of dominant species in non-grazed (NG) and over-grazed (OG) plots in the seven grassland types. Values are Mean±SE. A, B, PG, PF, S, and SS represent annuals, biennials, perennial grasses, perennial forbs, shrubs and sub-shrubs, respectively.

Species	Photosynth. Path way	Life Form	δ^{13}C Value (‰)
**Plain Meadow**			
NG Plot			
Allium tenuissimum	C_3	PG	-25.40±0.08
Artemisia eriopoda Bge.	C_3	PG	-28.33±0.10
Artemisia mongolica (Fisch. ex Bess.) Nakai	C_3	PG	-27.94±0.07
Artemisia scoparia Waldst. et Kit.	C_3	PG	-26.43±0.07
Cleistogenes squarrosa (Trin.) Keng	C_4	PG	-14.87±0.15
Cynanchum chinense R. Br.	C_3	PG	-26.20±0.31
Hedysarum gmelinii	C_3	PG	-26.83±0.13
Hemarthria altissima (Poir.) Stapf et C. E. Hubb.	C_4	PG	-12.88±0.09
Heteropappus altaicus (Willd.) Novopokr	C_3	PG	-28.24±0.13
Inula helenium Linn.	C_3	PG	-28.05±0.13
Leontopodium leontopodioides (Willd.) Beauv.	C_3	PG	-28.23±0.04
Lespedezadavurica(Laxm.)Schindl.	C_3	SS	-27.64±0.13
Leymus chinensis (Trin.) Tzvel.	C_3	PG	-26.41±0.11
Melissilus ruthenicus(L.)Peschkova	C_3	PG	-26.70±0.03
Oxytropis fetissovii Bunge	C_3	PG	-26.60±0.09
Oxytropis leptophylla (Pall.) DC.	C_3	PG	-26.76±0.07
Pedicularis flava Pall.	C_3	PG	-27.40±0.05
Potentilla conferta Bge. var. conferta	C_3	PG	-27.28±0.05
Puccinellia sibirica Holmb.	C_3	PF	-30.38±0.12
Serratula centauroides Linn.	C_3	PG	-28.98±0.13

Setaria viridis (L.) Beauv.	C_4	A	-15.13±0.18
Vicia amoena Fisch. ex DC.	C_3	PG	-26.92±0.07
OG Plot			
Chloris virgata Sw.	C_4	A	-13.57±0.21
Cynanchum chinense R. Br.	C_3	PG	-28.37±0.08
Leymus chinensis (Trin.) Tzvel	C_3	PG	-24.59±0.02
Melissilus ruthenicus(L.)Peschkova	C_3	PG	-27.03±0.12
**Meadow Steppe**			
NG Plot			
Agrostis alba L	C_3	PG	-28.86±0.02
Alisma plantago-aquatica L.	C_3	PG	-27.70±0.75
Carex duriuscula C. A. Mey.	C_3	PG	-29.83±0.06
Echinochloa caudata Roshev.	C_4	PG	-11.33±0.09
Juncus bufonius Linn.	C_3	PG	-28.76±1.52
Melissilus ruthenicus(L.)Peschkova	C_3	PG	-29.43±0.03
Plantago asiatica L.	C_3	PG	-29.38±0.03
Polygonum hydropiper L.	C_3	PG	-30.68±0.05
Potentilla anserina L.	C_3	PG	-29.23±0.04
Schoenoplectus tabernaemontani (C. C. Gmelin) Palla	C_3	PG	-28.80±0.02
Sium suave Walt.	C_3	PG	-28.47±0.95
OG Plot			
Artemisia frigida Willd.	C_3	PG	-29.00±0.04
Cleistogenes squarrosa (Trin.) Keng	C_4	PG	-19.42±0.67
Leontopodium leontopodioides (Willd.) Beauv.	C_3	PG	-28.97±0.05
Potentilla acaulis L.	C_3	PG	-28.51±0.11
Stellera chamaejasme L.	C_3	PG	-27.47±0.02
Thymus mongolicus Ronn	C_3	PG	-29.07±0.05
**Typical Steppe**			

NG Plot

Agropyron cristatum (L.) Gaertn.	C_3	PG	-25.98±0.17
Allium senescens	C_3	PG	-25.15±0.12
Allium tenuissimum	C_3	PG	-26.30±0.06
Artemisia frigida Willd.	C_3	PG	-27.83±0.09
Artemisia scoparia Waldst. et Kit.	C_3	A	-25.63±0.10
Calamagrostis epigeios (L.) Roth	C_3	PG	-25.72±0.07
Cannabis sativa L.	C_3	A	-25.69±0.12
Caragana microphylla Lam.	C_3	S	-24.58±0.09
Chenopodium glaucum L.	C_3	A	-26.08±0.02
Cleistogenes squarrosa (Trin.) Keng	C_4	PG	-14.48±0.10
Fallopia convolvulus (L.) Love	C_3	B	-25.28±0.04
Kochia prostrata (L.) Schrad.	C_4	SS	-14.30±0.03
Lappula myosotis V. Wolf	C_3	PG	-25.33±0.20
Poa annua L.	C_3	A	-24.72±0.04
Potentilla acaulis L.	C_3	PG	-27.02±0.18
Potentilla bifurca L	C_3	PG	-24.83±0.05
Potentilla tanacetifolia Willd. ex Schlecht.	C_3	PG	-25.90±0.09
Salsola collina Pall.	C_3	A	-25.76±0.07
Saposhnikovia divaricata (Trucz.) Schischk.	C_3	B	-27.37±0.20
Stipa krylovii Roshev.	C_3	PG	-24.16±0.06
Thalictrum simplex L.	C_3	PG	-27.69±0.02
Agropyron cristatum (L.) Gaertn.	C_3	PG	-26.81±0.15
Allium senescens	C_3	PG	-28.49±0.05
Artemisia frigida Willd.	C_3	PG	-27.04±0.04
Artemisia scoparia Waldst. et Kit.	C_3	B	-26.79±0.12
Artemisia scoparia Waldst. et Kit.	C_3	B	-26.22±0.23
Astragalus melilotoides Pall.	C_3	PG	-25.68±0.12

Lightly grazed (LG) plot			
Caragana microphylla Lam.	C_3	S	-25.21±0.20
Carex duriuscula C. A. Mey.	C_3	PG	-24.46±0.14
Heteropappus altaicus (Willd.) Novopokr.	C_3	PG	-27.47±0.17
Kochia prostrata (L.) Schrad.	C_4	SS	-15.33±0.03
Leymus chinensis (Trin.) Tzvel.	C_3	PG	-26.18±0.15
Limonium bicolor (Bag.) Kuntze	C_3	PG	-29.14±0.10
Potentilla acaulis L.	C_3	PG	-28.84±0.08
Potentilla bifurca L	C_3	PG	-26.09±0.08
Potentilla tanacetifolia Willd. ex Schlecht.	C_3	PG	-27.97±0.06
Saposhnikovia divaricata (Trucz.) Schischk.	C_3	B	-27.34±0.08
Spiraea aquilegifolia Pall.	C_3	S	-27.01±0.03
Stipa capillata L.	C_3	PG	-24.74±0.36
Urtica cannabina L.	C_3	PG	-23.52±0.07
OG Plot			
Agropyron cristatum (L.) Gaertn	C_3	PG	-28.07±0.06
Allium senescens	C_3	PG	-25.86±0.03
Artemisia frigida Willd.	C_3	PG	-28.49±0.09
Caragana microphylla Lam.	C_3	S	-26.78±0.11
Carex duriuscula C. A. Mey.	C_3	PG	-26.74±0.14
Chenopodium album L.	C_3	A	-26.46±0.10
Chenopodium aristatum Linn.	C_3	A	-26.96±0.07
Cymbaria dahurica Linn.	C_3	PG	-27.90±0.14
Cynanchum thesioides (Freyn) K. Schum.	C_3	PG	-27.05±0.04
Dontostemon dentatus (Bunge) Lédeb.	C_3	PG	-25.28±0.03
Heteropappus altaicus (Willd.) Novopokr	C_3	PG	-26.71±0.07
Kochia prostrata (L.) Schrad.	C_4	SS	-15.01±0.22
Leonurus artemisia (Laur.) S. Y. Hu	C_3	B	-25.66±0.18

Leymus chinensis (Trin.) Tzvel.	C_3	PG	-25.24±0.11
Potentilla acaulis L.	C_3	PG	-28.95±0.03
Potentilla bifurca L	C_3	PG	-26.02±0.09
Serratula centauroides Linn.	C_3	PG	-27.99±0.03
Spiraea japonica Linn. f.	C_3	S	-26.47±0.04
Stipa capillata L	C_3	PG	-26.63±0.12
Thalictrum simplex L.	C_3	PG	-26.86±0.06
Thalictrum squarrosum Steph.	C_3	PG	-27.53±0.06
**Desert Steppe**			
NG Plot			
Agropyron cristatum (Linn.) Gaertn.	C_3	PG	-25.03±0.11
Allium mongolicum Regel	C_3	PG	-26.05±0.12
Artemisia frigida Willd	C_3	PG	-25.51±0.02
Astragalus membranaceus (Fisch.) Bunge	C_3	PG	-24.54±0.05
Calystegia sepium (Linn.) R. Br.	C_3	PG	-25.01±0.04
Caragana microphylla Lam.	C_3	S	-23.91±0.07
Cleistogenes squarrosa (Trin.) Keng	C_4	PG	-15.14±0.23
Heteropappus altaicus (Willd.) Novopokr	C_3	PG	-26.07±0.04
Kochia prostrata (Linn.) Schrad.	C_4	SS	-15.59±0.02
Leymus chinensis (Trin.) Tzvel	C_3	PG	-24.89±0.06
Stipa capillata Linn.	C_3	PG	-23.57±0.07
OG Plot			
Agropyron cristatum (Linn.) Gaertn.	C_3	PG	-25.92±0.05
Artemisia frigida Willd	C_3	PG	-27.18±0.04
Calystegia sepium (Linn.) R. Br.	C_3	PG	-26.66±0.05
Cleistogenes squarrosa (Trin.) Keng	C_4	PG	-15.39±0.07
Heteropappus altaicus (Willd.) Novopokr	C_3	PG	-26.97±0.07
Kochia prostrata (Linn.) Schrad.	C_4	SS	-14.37±0.06

Leymus chinensis (Trin.) Tzvel	C_3	PG	-23.35±0.02
Potentilla bifurca Linn.	C_3	PG	-26.92±0.09
Potentilla strigosa Pall. ex Pursh	C_3	PG	-27.86±0.09
Stipa capillata Linn.	C_3	PG	-24.68±0.17
**Temperate Marsh Steppe**			
NG Plot			
Agrostis matsumurae Hack. ex Honda	C_3	PG	-25.52±0.03
Apium graveolens Linn	C_3	PG	-27.53±0.06
Caltha palustris L.	C_3	PG	-27.48±0.09
Carex duriuscula C. A. Mey.	C_3	PG	-26.31±0.07
Potentilla anserina L.	C_3	PG	-28.04±0.03
Potentilla discolor Bge.	C_3	PG	-28.14±0.05
Sanguisorba tenuifolia Fisch. ex Link var. alba Trautv.et Mey.	C_3	PG	-27.69±0.06
OG Plot			
Carex duriuscula C. A. Mey.	C_3	PG	-27.42±0.06
Elymus nutans Griseb.	C_3	PG	-26.50±0.03
Foeniculum vulgare Mill.	C_3	PG	-28.11±0.06
Plantago depressa Willd.	C_3	B	-27.65±0.05
Poa annua L.	C_3	A	-26.36±0.03
Potentilla anserina L.	C_3	PG	-28.25±0.04
Potentilla bifurca Linn.	C_3	PG	-27.82±0.05
Taraxacum mongolicum Hand.-Mazz.	C_3	PG	-26.75±0.06
**Alpine Meadow**			
NG Plot			
Androsace tapete Maxim.	C_3	PG	25.77±0.04
Aster alpinus Linn.	C_3	PG	-25.79±0.03
Astragalus membranaceus (Fisch.) Bunge	C_3	PG	-25.94±0.07

Bangia atropurpurea	C_3	A	-26.08±0.10
Comastoma polycladum (Diels et Gilg) T. N. Ho	C_3	A	-27.29±0.08
Elymus nutans Griseb.	C_3	PG	-25.94±0.15
Gentiana macrophylla Pall.	C_3	PG	-25.79±0.02
Gentiana sino-ornata Balf. f.	C_3	PG	-26.50±0.03
Heracleum dissectifolium K. T. Fu	C_3	PG	-25.69±0.03
K.humilis	C_3	PG	-26.88±0.06
Kobresia littledalei C. B. Clarke	C_3	A	-26.47±0.02
Kobresiapygmaea	C_3	A	-25.68±0.05
Leontopodium nanum (Hook. f. et Thoms.) Hand.-Mazz.	C_3	PG	-27.33±0.02
Oxytropis deflexa (Pall.) DC.	C_3	PG	-24.98±0.06
Oxytropis ochrocephala Bunge	C_3	PG	-25.64±0.09
Polygonum viviparum Linn.	C_3	PG	-25.69±0.05
Potentilla anserina L.	C_3	PG	-28.18±0.03
Stipa breviflora Griseb	C_3	PG	-25.36±0.10
Taraxacum mongolicum Hand.-Mazz.	C_3	PG	-26.09±0.03
OG Plot			
Ajania tenuifolia (Jacq.) Tzvel.	C_3	PG	-27.79±0.04
Androsace tapete Maxim.	C_3	PG	-25.06±0.27
Astragalus polycladus Bur. et Franch.	C_3	PG	-25.96±0.08
Comastoma polycladum (Diels et Gilg) T. N. Ho	C_3	A	-28.11±0.03
Gentiana macrophylla Pall.	C_3	PG	-24.19±0.12
Kobresiapygmaea	C_3	A	-26.04±0.09
Lagotis brachystachya Maxim.	C_3	PG	-26.23±0.04
Leontopodium nanum (Hook. f. et Thoms.) Hand.-Mazz.	C_3	PG	-27.56±0.01
Ligularia virgaurea (Maxim.) Mattf.	C_3	PG	-28.18±0.04
Oxytropis ochrocephala Bunge	C_3	PG	-25.55±0.03
Potentilla discolor Bge.	C_3	PG	-27.28±0.02

Saussurea pulchra Lipsch.	C_3	PG	-26.48±0.06
Saussurea stella Maxim.	C_3	PG	-25.65±0.08
**Temperate Marsh Steppe**			
NG Plot			
Androsace tapete Maxim.	C_3	PG	-29.11±0.05
Artemisia tanacetifolia	C3	PG	-26.54±0.08
Heteropappus altaicus (Willd.) Novopokr	C_3	PG	-26.12±0.04
Leontopodium leontopodioides (Willd.) Beauv.	C_3	PG	-29.23±0.01
Melissilus ruthenicus(L.)Peschkova	C_3	PG	-26.07±0.01
Phlomis mongolica Turcz.	C_3	PG	-28.48±0.09
Thymus mongolicus Ronn	C_3	PG	-28.49±0.03
OG Plot			
Androsace tapete Maxim.	C_3	PG	-27.93±0.11
Artemisia tanacetifolia	C_3	PG	-26.69±0.02
Artemisia tanacetifolia	C3	PG	-26.54±0.08
Astragalus membranaceus (Fisch.) Bunge	C_3	PG	-27.79±0.11
Festuca ovina L.	C_3	PG	-26.77±0.04
Heteropappus altaicus (Willd.) Novopokr	C_3	PG	-28.71±0.07
Rheum palmatum L.	C_3	PG	-26.05±0.04
Stipa capillata L.	C_3	PG	-26.7±0.07
Thymus mongolicus Ronn	C_3	PG	-28.10±0.03

Table S2 | The δ^{13}C values of species that showed converted photosynthetic pathway. Values are Mean±SE. NG and OG represent non-grazed and overgrazed treatment, respectively.

Steppe Type	Species	NG		OG	
		δ^{13}C value (‰)	Photosynth. Pathway	δ^{13}C value (‰)	Photosynth. Pathway
Plain meadow	*Phragmites australis (Cav.) Trin. ex Steud*	-26.55±0.14	C_3	-13.75±0.12	C_4
Typical steppe	*Pennisetum centrasiaticum Tzvel.*	-25.11±0.41	C_3	-14.74±0.08	C_4
Typical steppe	*Salsola collina Pall.*	-25.76±0.07	C_3	-13.84±0.24	C_4

Table S3 | The intercellular CO_2 concentration (*Ci*) values of two dominate species. Values are Mean ± SE (μmol/mol), NG and OG represent non-grazed and overgrazed treatment, respectively.

Steppe Type	Species	NG	OG	Referance
Desert steppe	*Leymus chinensis (Trin.) Tzvel.*	380 ±3	390 ±14	This study
	Stipa capillata L.	383 ±8	384 ±28	
Tipical steppe	*Leymus chinensis (Trin.) Tzvel.*	211 ±22	229 ±27	S. P. Chen[26]
	Stipa capillata L.	217 ±9	230 ±16	

Reference

26. Chen, S. P. *et al.* Effects of grazing on photosynthetic characteristics of major steppe species in the Xilin River Basin, Inner Mongolia, China. *Photosynthetica*. **43**. 559-565 (2005).
